# Ornamental Flowers Grown in Human Surroundings as a Source of Anthocyanins with High Anti-Inflammatory Properties

**DOI:** 10.3390/foods11070948

**Published:** 2022-03-25

**Authors:** Grzegorz P. Łysiak

**Affiliations:** Department of Ornamental Plants, Dendrology and Pomology, Poznan University of Life Sciences, ul. Dąbrowskiego 159, 60-594 Poznań, Poland; glysiak@up.poznan.pl

**Keywords:** agroecosystem, urban ecology, perennial and annual flowers, anti-inflammatory, antioxidant properties, biological activity, simple processing, post-harvest

## Abstract

Flowers have always accompanied people thanks to their manifold aesthetic properties. Some species have also become a component of the human diet. Recent years have seen an increased interest in edible flowers and, consequently, research has been undertaken to determine their chemical composition. Dyes that are abundantly contained in flowers, whose role is to attract pollinating animals, are recognized substances with health-promoting properties. Anthocyanins are a group of dyes that are very common in petals and other parts of flowers. Studies carried out in the twentieth and twenty-first century on flowers growing in temperate climates have found very strong antioxidant and anti-inflammatory properties of anthocyanins. Therefore, flowers used by humans for centuries to decorate their surroundings may become an easily available source of nutrients and health-promoting substances. This paper discusses the health-promoting properties of anthocyanins and collects literature on anthocyanin content in edible flowers commonly grown on balconies, terraces, and roofs in countries of temperate climate.

## 1. Introduction

An improved quality of life, including better nutrition, has significantly improved the life expectancy of the world population. This tendency is especially visible in western countries, but at the same time, the high availability of hypercaloric food and the increase in consumption of highly processed foods have led to the massive occurrence of chronic non-communicable diseases—mainly cardiovascular, metabolic and neuro-degenerative diseases in those countries [1]. In 2016, the World Health Organization (WHO) estimated that approximately 650 million adults were obese [2]. For these reasons, particular emphasis should be placed on increasing the consumption of fresh food containing bioactive compounds, as these substances provide health protection when interacting at many levels. Among fresh foods, plant-based foods especially contain a lot of bioactive compounds, such as polyphenolic compounds, which modulate processes occurring in the human body and have antioxidant, anti-inflammatory, anticancer, and neuroprotective effects, and they can modulate glucose levels [3].

The growing demand for new nutraceutical plant food has sparked interest in edible flowers. Various flower pigments, formed in the process of evolution to attract pollinator organisms, have been shown to have high antioxidant capacity, which can be a remedy for diseases of civilization [4]. Anthocyanins play an important role in the attraction strategy involving the use of color, but their strong antioxidant potential also makes flowers an important resource, the use of which should be increased in cultivation and nutrition [5]. Flowers commonly grown by humans, and thus often present in the human environment (for example, planted every year in containers on balconies, terraces, and roofs) due to the high content of biologically active substances, could become something of a “home pharmacy” helping to fight modern diseases.

## 2. Chemistry and Biochemistry of Anthocyanins

The word ‘anthocyanin’ derives from two Greek words: anthos, which means flowers, and kyanos, which means dark blue [6].

Anthocyanins are secondary metabolites in land plants that contribute to the color of leaves and flowers [7]. These pigments are primary blue, red, and purple. They are synthesized via the flavonoid pathway, which is part of the general phenylpropanoid pathway [8]. The entry to the biosynthesis of phenylpropanoids is the shikimate pathway. In this pathway, plants biosynthesize, in three steps, hydroxycinnamic acids and their derivatives, which are the precursors for a large variety of aromatic metabolites [9]. The next step before the synthesis of anthocyanidins is the conversion of a chorismic acid to phenylalanine by the enzyme phenylalanine ammonia-lyase [10], from which cinnamic acid is formed. The conversion of cinnamic acid to anthocyanins requires a series of reactions: the first reaction is catalyzed by cinnamate 4-hydroxylase to form a coumaric acid and by 4-hydroxy-cynnamoyl CoA ligase to create 4-Coumaroyl CoA, which is a direct precursor to kaempferol. After four steps of enzymatic reaction from 4-Coumaroyl CoA, the leucoanthocyanidins are formed [11]. By the catalysis of anthocyanin synthase (ANS), the colorless leucoanthocyanidins (flavan-3,4-diols) are oxidized to the colored anthocyanidins [9]. Flavan-3,4-diols, also known as leucoanthocyanidins, are not particularly prevalent in the plant kingdom, instead being themselves precursors of flavan-3-ols (catechins), anthocyanidins, and condensed tannins (proanthocyanidins). Anthocyanidins are unstable under physiological conditions, so they are immediately glycosylated in the 3-OH positions by UDP-glucose-flavonoid 3-O-glucosyltransferase (UFGT) to form the more hydrophilic and stable anthocyanins [12].

It has been experimentally demonstrated that all anthocyanin pigments are derived from one of three aglycones (pelargonidin, cyanidin, and delphinidin). The differences in the color of anthocyanins result from the pattern of hydroxylation and methylation and the amount and type of sugars [13]. Anthocyanins display different colors (red, blue, or purple) depending on their accumulation and chlorophyll complementary light absorbance. At low pH values, anthocyanins are present as flavylium cations (oxonium charged oxygen), while under neutral conditions, uncharged quinones are formed [14]. At a pH around 2.0–3.5, anthocyanins have a pink-coral color, while at 5.5–6.5, they are blue to purple [13]. The chromophore of conjugated double bonds carrying a positive charge on the heterocyclic oxygen ring is responsible for the intense red-orange to blue-violet color produced by anthocyanins under acidic conditions [15].

There were 635 identified anthocyanins in 2010 [16]. Anthocyanins are present in nature mainly in the form of heterosides. The aglycon form of anthocyanins are called anthocyanidin. The basic structure of anthocyanins is composed of flavylium cation (C6-C3-C6), which could be linked to different sugars or hydroxyl or methoxyl groups [17]. The most abundant anthocyanins are delphinidin, cyanidin, petunidin, peonidin, malvidin, and pelargonidin. Glucose is the most common sugar attached to anthocyanins, but rhamnose, xylose, galactose, arabinose, and rutinose have also been reported to be linked to these compounds [6]. Depending on the number of attached sugars, anthocyanins can be mono-, di-, or tri-glycosides [17]. The presence of sugars gives more stability and water solubility than their corresponding glycosides [9]. Glycosylation, primarily at the C-3 residue, results in reduced maximum wavelength absorption [18].

Sugar residues may be further acylated with cinnamic acids, such as p-coumaric, ferulic, and sinapic acid, as well as aliphatic acids, such as acetic, malonic, and oxalic acid [19].

## 3. Antioxidant Capacity and Anti-Inflammatory Property of Anthocyanins

### 3.1. Anioxidant Activity

The antioxidant potential of anthocyanins depends on the ring orientation (which determines the ease with which a hydrogen atom from a hydroxyl group can be donated to a free radical), the ability of the anthocyanin to support an unpaired electron [20], the number of free hydroxyls around the pyrone ring and their positions, and the presence of other types of radicals in the main structure [21]. The protection of these pigments against the oxidation process depends on their structures. Principally, the antioxidant capacity of anthocyanins is associated with the number of free hydroxyls around the pyrone ring. Higher antioxidant capacity is due to the number of hydroxyls [20].

Individual anthocyanins differ in their ability to remove highly active radicals depending on the radical. For instance, pelargonidin is the most efficient against the hydroxyl radical, whereas delphinidin is the most active against the superoxide anion [22]. Free radical damage contributes to the etiology of many chronic diseases, and thus, antioxidants may have beneficial effects on human health at different levels [23]. Improving the diet through the consumption of products containing natural antioxidants is one of the best strategies to create a balance between the activity of free radicals and the antioxidant system in the human body [24].

The antioxidant capacity of consumed products can be measured using chemical, in vitro methods generally performed on extracts. The literature mentions nearly 20 methods [9,11,13], but in general, if there are many methods, none of them is perfect. In addition, we must remember that the indicators give us a picture of the potential of the product, but they will not answer the question of how many substances will be absorbed and what impact they will have on the body. The most popular methods of measuring antioxidant capacity are based on the ability to bind free radicals (DDPH, ABTS), to reduce cupric or ferric ions (FRAP, CUPRAC), to protect a target molecule exposed to a free radical source (ORAC, TRAP), and to inhibit the oxidation of low-density lipoprotein (LDL) [25].

Antioxidant capacity is a function of the content and types of phytochemicals that are present in fresh tissues. However, individual groups of compounds may differ considerably in terms of antioxidant capacity. Many studies indicate that phenols and flavonoids contribute more strongly to antioxidant capacity than ascorbic acid, vitamins, carotenoids, and other compounds [26]. Anthocyanin molecules, due to their structure, stand out from flavonoids as a group of compounds exhibiting very high antioxidant capacity [27]. Still, some research suggests that the bioavailability of anthocyanins is lower than that of other flavonoids. Anthocyanins were initially perceived as poorly absorbed and metabolized compounds, which cast doubt on whether they could have a biological effect in humans. They were found only in the plasma in their intact form (glycosylated). However, most of those studies were based on plasma and urine analysis for anthocyanin metabolites derived from glucuronidation and sulphation metabolism. More recent studies increasingly allow to identify metabolites of anthocyanins at higher concentrations than the parent compounds [28]. According to some research, anthocyanins may be metabolized by intestinal microflora, producing a group of new products that have not yet been identified, not to mention quantified. In addition, recent studies indicate that anthocyanins are rapidly absorbed, with a maximum plasma concentration (Cmax) between 45 min to 4 h after ingestion of a meal containing anthocyanins, depending on the conditions of the trial. When anthocyanins were ingested alone and after a night, Cmax was reached after only 1 h [29], but if they were consumed together with other food, the absorption decreased; especially if food contained fat, Cmax was reached only after 4 h [30]. The structure of anthocyanins affects their absorption by the human body. It has been shown that 3-monoglucosides of anthocyanidins are less bioavailable than their corresponding rutinosides [31]. The absorption differences between malvidin and petunidin may also be due to the fact that a large number of hydroxyl groups in the molecule decreases its bioavailability. However, it should be remembered that the absorption capacity will also depend on the number of anthocyanins and the presence of other compounds. It was found out that the ingestion of anthocyanins together with sugar slowed down their absorption, while the consumption of anthocyanins together with alcohol significantly accelerated their intake [32].

### 3.2. Anti-Inflamtory Activity and Protection against Chronic Diseases

The health benefits of anthocyanins have been studied in a variety of models, ranging from human clinical trials to animal and cell culture screening to epidemiological studies [17]. The human body is in constant contact with external factors that can cause various types of damage, irritation, or allergies [9], often leading to inflammation. Inflammation is a complex set of relationships between soluble compounds that can arise in any tissue in defensive response to traumatic, infectious, post-ischemic, toxic, or autoimmune injury. It is typically induced by microbial infections but can also be triggered by tissue injury or trauma that occurs without the intervention of pathogens (sterile inflammation). The inflammation process usually leads to recovery from infection and healing [33].

Adaptive innate immune response induces rapid activity following infection. A wide range of molecular patterns are detected, commonly found in pathogens but are foreign to mammals. They are called pathogen-related molecule patterns (PAMP) [34]. Such particles are lipopolysaccharides, surface phosphatidylserine, and aldehyde derivatized proteins, as well modified forms of the classic risk factor for atherosclerosis, oxidatively modified low-density lipoprotein (LDL), or glycation [35]. The cellular response may be lysosomal endocytosis, degradation-bound ligands. Involvement in the process of Toll-like receptors causes the activation of the nuclear factor kappa B (NF-κB) and protein kinase. It can induce increased phagocytosis, production of reactive oxygen, and release of cytokines, autacoids, and lipids coordinating and strengthening local inflammation [36,37]. Recent research demonstrates that metabolites of anthocyanins can reduce the activation of NF-κB [38]. Protein kinases, cellular stress kinases, extracellular signal-regulated kinases, and mitogen-activated protein kinases (AMPK) are other molecular targets of anthocyanins and have been shown to be sensitive to anthocyanin treatment, reducing downstream cellular signaling networks associated with serious diseases, such as chronic inflammation [17]. AMPK-activated protein kinase involved in cellular energy (glucose) metabolism-caused diabetes appears to be one of the main targets of anthocyanins [39]. AMPK is an important regulator of energy homeostasis and is a molecular target of drugs used for the treatment of obesity and other metabolic diseases [40]. Another target of anthocyanins are thrombin receptor-activating peptide and vascular endothelial growth factor, which are responsible for angiogenesis, cancer, and atherosclerotic risk [41].

The biological activity of isolated anthocyanins and anthocyanidins, or foods rich in anthocyanins, can be manifested in the prevention of cardiovascular disease [42], influence on cholesterol distribution, protection of endothelial cells from CD40-induced proinflammatory signaling [43], anticancer, antitumor, and antimutagenic activity [44], beneficial effects in diabetes [45], protective effects against oxidative liver damage [46], protective effects on gastric inflammation and damage [47], antimicrobial and antiviral activity [48,49], slowing down neuronal and behavioral aging [50], and protection from some neurodegenerative diseases such as Alzheimer’s disease [51]. Anthocyanins and anthocyanidins also effectively induce insulin secretion when tested in pancreatic cell lines [11]. The effectiveness of insulin secretion depends on the number of hydroxyl groups in the B-ring of their structures [45].

Cyanidin (C_15_H_11_O_6_) and its derivatives are the most common anthocyanins in flowers (Table 1). The study carried out by Samarpita and Rasool [52] suggests that cyanidin is a potent inhibitor of Interleukin (IL)-17A signaling associated with the pathogenesis of rheumatoid arthritis, the most common autoimmune arthropathy. Cyanidin not only effectively blocks interleukin 17A/p38 but also suppresses osteoclastogenesis. This study suggests that cyanidin has great potential as a small molecule drug to be used in clinics to treat rheumatoid arthritis patients [52]. Moreover, there is evidence that cyanidin as well as delphinidin have the chemo preventive effect against skin cancer [53].

The effect of anthocyanins on microbial pathogens has not been studied in depth up to now. However, the results obtained so far are very promising.

## 4. Factors Influencing Anthocyanin Content in Ornamental Plants

Anthocyanin accumulation is strongly regulated by plant development and genotype and by environmental factors [96]. One of the goals of ornamental plant breeding is to broaden the color palette of different species by adding missing colors. For example, the best-selling cut flowers so far are, namely, rose, chrysanthemum, carnation, and lily, including no blue cultivars in their palette, while petunias are not red or orange [97]. Purple flowers in rose and carnation were obtained by changing the decoration pattern on the basic skeleton of anthocyanins, i.e., increasing the accumulation of delphinidin [98].

Temperature has a big impact on anthocyanin accumulations. Strong temperature variations between day time and night time favor the accumulation of soluble solids, and more soluble solids enhance the accumulation of anthocyanin [99]. However, too low temperatures slow down physiological processes and can thus also limit anthocyanin production. The plant hormone abscisic acid (ABA) has been suggested to play an important role in anthocyanin accumulation. ABA treatment increases anthocyanin content in grape skin and induces the expression of anthocyanin-biosynthesis genes [100]. Studies on the effect of altitude on anthocyanin accumulation in blueberry fruit found out that plants growing at a lower altitude accumulated more anthocyanins [96].

Fertilization also affects anthocyanin accumulations. Pre-harvest calcium treatment was shown to upregulate the expression of anthocyanin structural genes and to increase the total phenolic and anthocyanin content [101]. The accumulation of anthocyanins in the plant is also promoted by better availability of phosphorus in soil [102] and by the application of melatonin (N-acetyl-5-methoxytryptamine). It is explained that melatonin is involved in the secondary metabolism, where it induces anthocyanin and flavonoid biosynthesis [103]. The plant’s growing location also plays a role because it has been shown that ultraviolet B emitted by the sun (wave length: 280–315 nm) promotes anthocyanin synthesis. This is the part of the radiation that is only partially absorbed by the ozone layer, and therefore, exposure to direct sunlight stimulates the formation of anthocyanins [104].

The production of anthocyanins in ornamental plant species is also enhanced by a change in the activity of flavonoid enzymes by gene modification. This method to increase the content of anthocyanin pigments was applied to petunias and torenias [105,106]. At the genetic level, gibberellins, which are regulators of growth and development, can also interact. It was found that, during the development of petunia flowers, gibberellin induced the expression of some genes such as those of chalcone synthase, chalcone isomerase, anthocyanidin synthase, and dihydroflavonol 4-reductase, which are responsible collectively for corolla pigmentation [107,108].

## 5. Anthocyanin Content of Domestic Grown Edible Flowers

**Figure 1:**
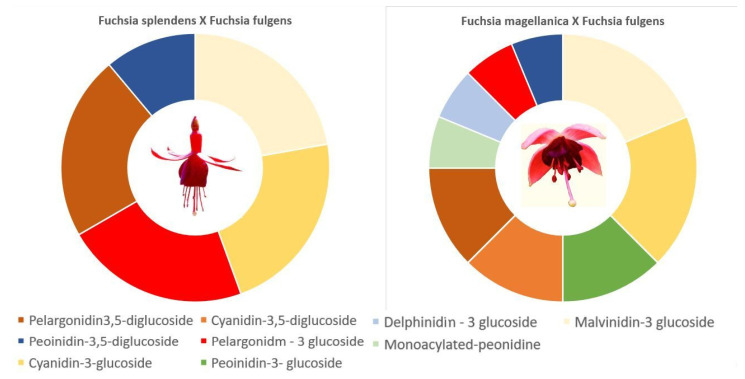
Distribution of anthocyanins in fuchsia hybrids [67,113,114].

The content of anthocyanins has not been tested yet in many edible flower species (Table 1 and Table 2). A number of studies were carried out in the 1980s and 1990s to find individual anthocyanins, sometimes also their derivatives [67,68,75,78]; however, there were no technical possibilities to allow the measurement of anthocyanin content. The highest total amount of anthocyanins was found in petals of perennial hibiscus; annual flowers with the highest anthocyanin concentration were *Dahlia* sp., *Petunia* sp., and *Tropaeolum majus* [62,79,80]. So far, it has been found that all species grown on balconies, terraces, or roofs of houses, whose flowers are edible, show high antioxidant capacity [5]. The content of anthocyanins strictly depends on the cultivar [78,80,109] and the development phase of flowers [90,109]. Annual flowers grown in the human environment can be a very important source of anthocyanins [58,64,85,110], but perennial flowers grown in larger pots, such as roses or hibiscus, are also known to be an excellent source of polyphenols [33,74,82,83]. Anthocyanin content can vary significantly both qualitatively and quantitatively, even in flowers of related species (Figure 1). Cultivars of the same species can also strongly differ in terms of anthocyanin content [82,83].

**Table 2:** Identified derivatives of anthocyanins listed in Table 1.

Species	Glycosileted and Acyleted Anthocyanins	Source
*Begonia*	Cyanidin 3-O-giucoside, pelargonidin 3-O-diglucoside, cyanidin 3-O-xylosylglucoside, cyanidin 3-O-rhamnosyldiglucoside Malvidin 3,5-diglucoside	[54,55]
*Bellis*	Cyanidin 3-malonylglucuronylglucoside Cyanidin 3-malonylglucoside, Pelargonidin 3-malonylglucoside Delphinidin 3-O-glucoside	[56,111]
*Campanula*	Pelargonidin 3-rutinoside-7-glucoside, Delphinidin 3-O-glucoside Cyanidin 3-glucoside, Cyanidin 3-rutinoside,	[59,112]
*Dahlia*	Cyanidin-rutinoside Pelargonidin 3,5-di-O-glucoside	[62]
*Dianthus*	Cyanidin 3-malylglucoside, Delphinidin 3-malylglucoside pelargonidin 3-malylglucoside	[63]
*Dendranthema*	Cyanidin 3-malonylglucoside Pelargonidin 3-O-glucoside, Delphinidin 3-O-glucoside)	[56] [65]
*Glechoma*	Cyanidin 3-(6”-maionylglucoside)~5-glucoside Delphinidin 3-(6”-pcoumarylgIucoside)-5-glucoside	[68]
*Hibiscus*	Cyanidin-3-sambubioside, Delphinidin-3-sambubioside.	[73]
*Impatiens*	Malvinidin glucoside, Pelargonidin glucoside Paeonidin glucoside	[75]
*Lobularia maritima*	Acylated pelargonidin 3-samububioside 5-glucoside cyanidin 3-sambubioside-5-glucosides	[76] [77]
*Pelargonium* spp.	Cyanidin 3,5-di-O-glucoside, Delphinidin 5-O-glucoside Pelargonidin 5-O-glucoside, Malvidin 3,5-di-O-glucoside Peonidin 3,5-di-O-glucoside, Petunidin 3,5-di-O-glucoside	[78]
*Petunia*	Cyanidin 3-O-rutinoside, Delphinidin 3-O-glucoside Pelargonidin 3-caffeoylrutinoside-5 rutinoside, Malvidin 3-caffeoylrutinoside, Peonidin 3-caffeoylrutinoside 3,5-di-O-glucoside, Petunidin 3 caumrylorutinoside 5-glucoside	[80]
*Rosa*	Cyanidin, Cyanidin-3-O-glucoside	[62]
*Tagetes erecta*	Cyanidin-di-hexoside, Delphinidin-3-*O*-hexoside	[84]
*Tagetes patula*	Cyanidin-3-galloylsophoroside, cyanidin-3-glucoside, cyanidin-3-sophoroside	[85]
*Tropaeolum majus*	Pelargonidin-3-*O*-sophoroside, delphinidin3-*O*-3-dihexosides; cyanidin3-*O*-sophoroside	[84,88]
*Torenia* sp.	Cyanidin 3,5-di-O-glucoside, Malvidin-3-O-β-D-glucoside,-Peonidin-3-glucoside-5-(p-coumaroyl)-glucoside	[86,87]
*Tulipa* sp.	Cyanidin 3-rutinoside, Delphinidin 3-rutinoside, Pelargonidin 3-rutinoside,,	[89]
*Viola cornuta*	Petunidin-O-deoxyhexoside- hexoside, Cyanidin 3-glucoside,	[93]
*Viola witrockiana*	Cyanidin-rhamnosyl-glucoside, Delphinidin-rhamnosyl-glucoside, yanidin-3-(coumaroyl)-methylpentosyl-hexosyl-5-hexoside	[94]

## 6. Pharmacy in the Neighborhood (Balconies, Roofs, Terraces)

We live in a world where only a small percentage of land remains relatively undisturbed. The urban landscape is not only meant to be functional, but also to actively provide cultural experiences and to create a harmonious structure [115]. Nowadays, terraces or residential courtyards in an urban or agricultural environment take up the role of kitchen gardens, contributing not only to the development of urban agriculture, but also to increasing the availability of health-promoting substances. People often turn balconies, roof terraces, or patios into an attractive space to dine and entertain with stylish lighting and furnishing ideas. At such “home plots” the cultivation of ornamental plants occupies an important place [116,117]. In addition to aesthetic advantages, such a location can also be a source of edible flowers, which, in addition to stunning delicacy, can perform their health-promoting functions. Flowers were already consumed in various European and Asian cultures as alternative medicines or as part of traditional food to improve nutritive value and/or the appearance of meals [118].

The ability of anthocyanins to induce antioxidant and detoxifying enzymes has potential implications for cancer prevention and for modifying cellular oxidant status [17]. Health and therapeutic effects of anthocyanins are related to their chemical and biochemical properties, which are partially explained by their antioxidant capacities. However, anthocyanins are relatively unstable and easily oxidized. They are sensitive to many factors, such as temperature, UV radiation, the presence of sulfur dioxide, and some ion’s ascorbic acid [106,119]. Therefore, easy access to edible flowers containing anthocyanins can support the bioavailability of these compounds to consumers. Cyanidin-3-glucoside, one of the most common anthocyanins in edible flowers, has the highest ORAC value; its Trolox value is 3.5-times the Trolox value of a water-soluble vitamin E analog.

The concentration of anthocyanins in fresh fruits and vegetables can significantly drop even during only several days of storage in a cold store, as demonstrated by a study on the level of pelargonidin 3-glucoside and cyanidin 3-glucoside, the two anthocyanidin glycosides responsible for the color of strawberries. Similar conclusions can be drawn based on various other studies, such as that on storing ‘Jonagold’ apples for 120 days, in which the amount of anthocyanins decreased during storage from 158 mg/100 g to 119 and 103 mg/100 g [120]. Growing flowers in direct neighborhood to home and harvesting them for direct consumption just before a meal makes it possible to avoid the degradation of these beneficial compounds.

## 7. Preservation and Simple Processing of Edible Flowers

Edible flowers are extremely perishable and delicate. Especially after harvest and during storage, flowers turn brown and wilt very quickly and are prone to fungal infection [121]. There are very few methods to keep harvested flowers fresh at home. The simplest method of extending the shelf life of all plant products (fruits, vegetables, herbs) is to slow down metabolic processes by lowering the temperature close to 0 °C or even slightly below [122,123]. This allows the storing of most flowers for up to two weeks [124].

The shelf life of fresh garden products can also be extended by changing the gas composition surrounding them. Limiting the access of oxygen with a simultaneous increase in the concentration of carbon dioxide slows down respiration [125], and thus visibly reduces the ripening ratio, perishability, and mold reproduction, and it decelerates decay [126,127]. This can be achieved by packaging fresh flowers in polyethylene bags or even better by using specially prepared boxes sealed with polymer film, preferably low-density polyethylene (LDPE) or polypropylene (BOPP) film, using a modified atmosphere packaging technology MAP [122,128,129]. Both LDPE and BOPP are soft, flexible, and strong with a good ratio of CO2 to O2 permeability and a good moisture barrier [130].

Another way to prolong the shelf life is to apply an edible coating [126]. This protects edible flowers from loss of moisture, anthocyanins, and other pigments, volatile, and flavor substances, and at the same time, it prevents odor absorption, oxidation, and enzymatic browning, and finally it inhibits or delays pathogenic infection [131,132]. Among various materials used to cover fruits and vegetables, only sodium alginate and chitosan have been used to coat edible flowers [132]. The aqueous solution of alginic acid salt can be used at home with very good results. It has been shown that alginate coating effectively delays the degradation of edible flowers stored in a refrigerator at 5 °C from 3–4 days up to 14 days [133].

Finally, edible flowers can be dried or frozen, but this preservation method deprives flowers of one of their basic advantages, namely, attractive appearance. In addition, anthocyanins and other biologically active compounds contained in them are partially decomposed, depending on the technology used [126].

## 8. Conclusions

The existing literature indicates that many ornamental plants growing in the immediate vicinity of humans can be an abundant source of anthocyanins. Many researchers focus on widely recognized products rich in anthocyanins, such as wine or berry plants. However, more studies are needed to determine which anthocyanins are present in edible flowers and at what concentrations. Biotechnology offers promising methods to increase anthocyanin levels in edible flowers, whereas more widespread cultivation of flowers in containers on balconies, terraces, and roofs makes it easier for humans to include them in their daily diet. The literature on the subject provides sufficient evidence showing that edible flowers rich in anthocyanins may have a protective effect on human health, especially by preventing cancer and neurodegenerative and cardiovascular diseases. Similar to fruits or vegetables, edible flowers are most attractive in terms of their health-promoting properties and appearance when they are fresh. Growing them in the immediate vicinity to home allows access to them for a large part of the year. In addition, there are methods that help keep them fresh at home for a certain period after harvest. Research on processing flowers as food components is focused on methods to maintain the highest possible level of biologically active compounds.

## Figures and Tables

**Table 1:** Anthocyanin content in edible flowers grown in containers.

Flower Species	Anthocyanins *								Source
	Cyanidin	Delphinidin	Pelargonidin	Malvidin	Peonidin	Petunidin	Total	ORAC/ FRAP ^1^ (TE/100 g) ^A^ (mmol FeSO_4_ /100 g) ^B^	
*Ageratum houstonianum*							27.85 ^8^	2.99 ^A,8^	[5],
*Argyranthemum* *houstonianum*							2.99 ^8^	27.85 ^B^	[5]
*Begonia* sp.	p ^D^		p ^D^	759.1			5.09 ^8^	21.18 ^B^	[5,54,55]
*Bellis perennis*	p ^D^		p ^D^				no data		[56]
*Campanula* sp.		p ^D^	p ^D^						[57,58]
*Calendula officinalis*							22.1 ^7^	3.68 ^A^ 58.05 ^B^	[5,59,60]
*Dahlia* sp.	121.2		2.65				17.6–257.5 ^7,9^	17–24 ^5,9^	[61,62]
*Dianthus*	52.4	p ^D^	p ^D^				0.73–13.35 ^8,9^	5.4–10.2 ^A,9^	[5,63,64]
*Dendranthema*	p ^D^	p ^D^	p ^D^					168–182 ^B^	[56,65,66]
*Phaseolus coccineus*									no data
*Fuchsia* sp.	p ^D^				p ^D^		7.58 ^8,9^	47.52 ^B^	[5,67]
*Glechoma hederacea*	p ^D^	p ^D^					no data		[68]
*Heliotropium oxalis*									no data
*Helichrysum*								419.8 ^B^	[69]
*Hemerocallis*								21.0–29.0 ^A^	[70]
*Hibiscus* sp.	2080	5650					155–206 ^8^	83.1 ^5^	[71,72,73,74]
*Impatiens*			p ^D^	p ^D^	p ^D^		no data		[75]
*Lavandula*								277.60 ^B^	[68]
*Lobelia*									no data
*Lobularia maritima*	p ^D^		p ^D^				no data		[76,77]
*Myosotis*								171.60 ^A^	[68]
*Pelargonium* spp.	p ^D^	p ^D^	p ^D^	p ^D^	p ^D^	p ^D^	12.52 ^8^	34.78 ^B,^^9^	[5,78]
*Petunia*	53.2 ^2^	31.3 ^2^	49.0 ^4^	2.6 ^4^	87.1 ^3^	8.5 ^3^	28–114 ^9^	5.4–10.22 ^B,9^	[5,79,80]
*Rosa*	357.0		31.2		140.4–153.1 ^10^		2.3–7.0 ^8^	71.4–397.4 ^A,9^	[58,62,81,82,83]
*Tagetes erecta*	33		3.8				0.75 ^8^	70.42 ^B^ 266.11 ^A^	[5,84]
*Tagetes patula*	0.25 ^6,9^	p ^D^		p ^D^		p ^D^		0.076–0.433 ^6,9^	[60,85]
*Torenia* sp.	0.9–41.0 ^9^	210.96		4.2–134.9 ^9^			5.0–152.7 ^9^	8907.50 ^A^	[55,86,87],
*Tropaeolum majus*	4.77 ^8^	32.208 ^8^	32.06 ^8^	–	–	–	68.12 ^8^	7111–18,719 ^A,9^	[88]
*Tulipa* sp.	p ^D^	p ^D^	p ^D^				3.8–4.0 ^9^	29.23 ^B,11^	[89,90,91,92]
*Viola cornuta*	70.0 ^7^	1350 ^7^						25.0 ^B^	[60,93]
*Viola wittrockiana*	1.9–16.7 ^9^	8.6–21.8 ^9^		8.8–14.2 ^9^		1.2–15. ^9^	0.35–13.6 ^9^	0.82–36.55 ^B,9^	[5,94,95]

* The content of individual anthocyanins is given together with their derivatives such as glycosides, rutinosides, and others, as a sum of identified anthocyanins, identified derivatives are listed in Table 2; ^1^ Oxygen radical absorbance capacity; ^D^ Presence identified but no quantitative data available; ^2^ Average value for 8 cultivars; ^3^ Average value for 3 cultivars; ^4^ Average value for 4 cultivars; ^5^ Antioxidant capacity was measured using 2, 2-diphenyl-1-picrylhydrazyl (DDPH), data expressed as percent inhibition of DPPH; ^6^ g/100 g FW, there is a sum of three cyanidins, the share of cyanidin-3-galloylsophoroside is 60–90%; ^7^ Equivalent of pelargonidin mg per gram of sample DW; ^8^ Equivalent of mg cy-3-glu/100 g FW and DW for Hibiscus, Petunia, Rosa, Tropaeolum; ^9^ Depends on the cultivar; ^10^ Depends on the season; ^11^ Wild species *Tulipa humilis*; ^A^ *ORAC*, ^B^ *FRAP*.

## Data Availability

Not applicable.
